# Case Report: Suspected Case of *Brucella*-Associated Immune Reconstitution Inflammatory Syndrome

**DOI:** 10.3389/fimmu.2022.923341

**Published:** 2022-07-22

**Authors:** Chunmei Qu, Nannan Xu, Dehong Niu, Sai Wen, Hui Yang, Shanshan Wang, Gang Wang

**Affiliations:** ^1^ Department of Infectious Disease, Qilu Hospital, Cheeloo College of Medicine, Shandong University, Jinan, China; ^2^ Department of Oncology, the Fifth People’s Hospital of Jinan, Jinan, China

**Keywords:** human brucellosis, IRIS, immune reconstitution, infection, case report

## Abstract

Human brucellosis is one of the most prevalent zoonoses. There are many similarities between the pathogenesis of *Mycobacterium tuberculosis* (MTB) infection and that of brucellosis. Immune reconstitution inflammatory syndrome (IRIS) may occur during the treatment of MTB infection, but it has not been reported in brucellosis cases thus far. We report the case of a 40-year-old male whose condition initially improved after adequate anti-*Brucella* therapy. However, 3 weeks later, the patient presented with exacerbation of symptoms and development of a paravertebral abscess. After exclusion of other possible causes of clinical deterioration, immune reconstitution inflammatory syndrome (IRIS) with brucellosis was presumed. After supplementation with anti-*Brucella* treatment with corticosteroids, the abscess disappeared, and the symptoms completely resolved. Our case suggests that it is necessary to be aware of the possible occurrence of IRIS in patients with brucellosis in clinical practice.

## Case Report

A 40-year-old male farmer without any underlying condition was admitted to the hospital because of fever, night sweats, and pain in the lower back. The patient had reportedly been well until 3 weeks earlier, when back pain developed. He reported no associated trauma or injury, and no treatment was administered. On the 10th day of illness, he began to have fever with a temperature as high as 38.3°C, along with night sweats, fatigue, and weakness. He took antipyretics, but the fever and back pain persisted. The patient was sent to this hospital for further evaluation. The patient had occupational exposure to livestock. He was married and lived with his wife and children, who were well. There was no history of tuberculosis (TB), recent travel, transfusions, alcohol consumption, smoking, or intravenous drug use. The patient took no medications and had no history of drug allergy. There was no family history of disease.

**Figure 1:**
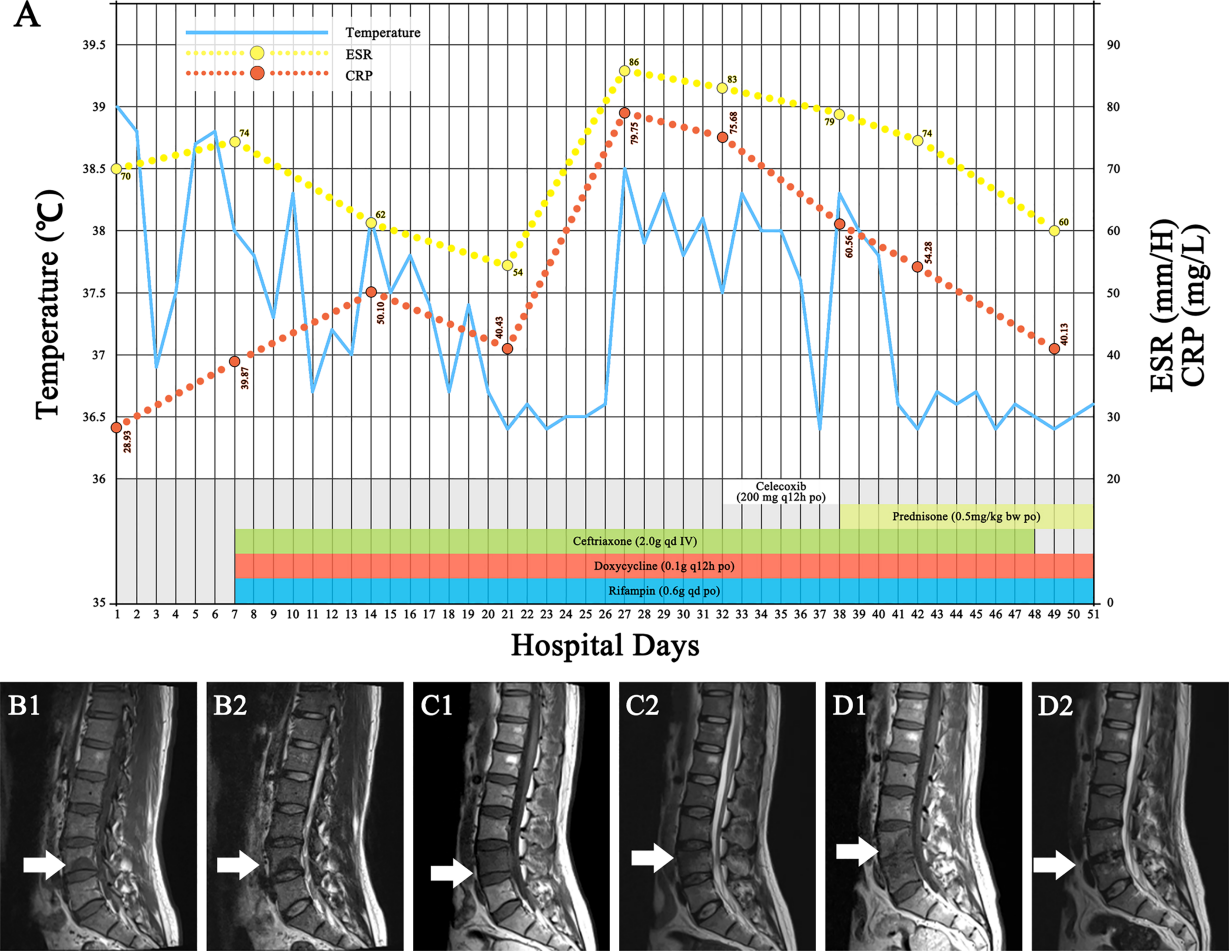
Clinical data. **(A)** The hospitalization course, with the timeline of antibiotic treatment and the changes in body temperature and inflammatory markers. **(B1, B2)** The first MRI scan of the patient showed a lesion on the lower ^1^/_2_ part of the fourth lumbar vertebra (white arrow), with low intensity on T1WI and high intensity on T2WI. **(C1, C2)** The second MRI scan, which occurred on follow-up day 27, showed that the lesion had expanded (white arrow), involving the posterior lower part of the fourth lumbar vertebra and with fusiform abscess formation behind the fourth vertebral body and lumbosacral soft tissue edema. **(D1, D2)** The last MRI scan (white arrow) after 3 months showed that the lower edge endplate of the fourth lumbar vertebra was damaged, the L4/L5 intervertebral disc was turbid, the retrovertebral abscess had disappeared, and the lumbosacral soft tissue edema was significantly improved. ESR, erythrocyte sedimentation rate; CRP, C-reactive protein.

Upon admission to the hospital, his core body temperature was 39°C, and he had severe pain in the L4–L5 area. Other vital signs and the remainder of the physical examination were normal. The erythrocyte sedimentation rate (ESR) was 70 mm/h. The levels of C-reactive protein (CRP) and interleukin-6 (IL-6) were slightly elevated to 28.93 mg/L and 10.88 pg/ml, respectively (**Figure 1A**). A human immunodeficiency virus (HIV) test was negative, and other blood parameters, including routine blood parameters, liver enzyme concentrations, procalcitonin and creatinine levels, and antinuclear antibody concentrations, were normal. Blood culture was negative. Magnetic resonance imaging (MRI) showed abnormalities suggesting inflammation in the L4 region (**Figure 1B**). An enzyme-linked immunosorbent assay (ELISA) for the detection of *Brucella* antibodies was performed on plasma, and the results were positive, with an IgM concentration of 12.88 U/ml and an IgG concentration of 89.15 U/ml. To confirm the diagnosis, vertebral tissue aspiration was performed on the second day after admission. The aspirate was sent for bacterial culture and molecular TB detection. An automated blood culture system was used for bacterial culture. Five days later, the bacterial culture result was positive for *Brucella*, which is a very small, faintly stained Gram-negative coccobacillus that microscopically looks like “fine sand”. Polymerase chain reaction (PCR) was negative for *Mycobacterium tuberculosis* (MTB) DNA. With a confirmed diagnosis of *Brucella*-related complicated infection, triple therapy including intravenous ceftriaxone (2.0 g qd) and oral rifampin (0.6 g qd) and doxycycline (0.1 g q12 h) was administered. After 2 weeks of treatment, the patient’s body temperature returned to normal. The pain in the lower back was also relieved. However, 1 week later (3 weeks from the beginning of anti-*Brucella* therapy), the patient’s symptoms recurred; he had a moderate to low fever (top temperature up to 38.5°C) accompanied by lower back pain. On physical examination, the pain in the L4–L5 area was significantly worse than before. The results of laboratory re-examination showed a normal white blood cell count (8.11 × 10^9^/L), with 62.1% neutrophils, and a highly elevated ESR (86 mm/h) and CRP level (79.75 mg/L). The level of IL-6 increased to 53.91 pg/ml. Liver enzymes, creatinine levels, and antinuclear antibodies were within normal ranges. The second MRI (**Figure 1C**) scan of the spinal cord showed lesion expansion involving the lower posterior part of L4 and focal abscess formation. Due to the recurrent clinical symptoms and imaging findings, abscess puncture and drainage were performed. Cytology showed inflammatory infiltration (of which 65% were neutrophils) without neoplastic cells. Abscess fluid culture results were negative. Despite drainage for 5 days, the symptoms of fever and lower back pain persisted.

Because the patient had been hospitalized since the beginning of treatment, poor treatment adherence could be excluded. Other infections and drug side effects were also ruled out. Accordingly, brucellosis-associated IRIS was suspected. There is no clinical consensus on the definition of infection-associated IRIS, and there is no treatment standard. With reference to the regimen for TB-IRIS treatment (1), the triple-agent anti-*Brucella* regimen was continued, and 200 mg of celecoxib twice a day was initiated. However, the patient’s symptoms remained after 1 week of treatment. Anti-inflammatory treatment was changed to 0.5 mg of prednisone per kilogram bodyweight (35 mg). Within 3 days, the patient’s body temperature returned to normal, and the back pain significantly improved. Both the ESR and CRP level also gradually returned to within normal ranges. Steroid therapy was tapered over a 2-month period. The triple-agent anti-*Brucella* therapy was continued for 2 weeks, followed by sequential treatment with oral doxycycline (0.1 g q12 h) and rifampin (0.6 g qd). After the overall 14-week treatment course, the third MRI scan (**Figure 1D**) showed that the lower edge endplate of L4 had been damaged, the paravertebral abscess had disappeared, and the lumbosacral soft tissue edema had significantly improved. At the last follow-up visit 2 months after completing the anti-*Brucella* therapy, the patient had no complaints, and the physical examination was normal.

## Discussion

IRIS is an excessive inflammatory response to infectious or noninfectious antigens after the reversal of underlying immunosuppression (2). The most common presentation is HIV-associated TB-IRIS (3), where patients’ symptoms worsen following the initiation of anti-retroviral therapy. It also occurs among HIV-uninfected patients (4–6). IRIS has also been observed in infections by other pathogens, such as *Mycobacterium leprae* (7), *Mycobacterium ulcerans* (8), the *Mycobacterium avium* complex, and *Cryptococcus* (9). There are two forms of IRIS: paradoxical and unmasking (1).

There are currently no definitive diagnostic criteria for IRIS, especially in HIV-uninfected patients. IRIS is a diagnosis of exclusion (1). In our case, this patient’s symptoms initially improved after adequate anti-*Brucella* treatment, but he subsequently presented with the paradoxical exacerbation of brucellosis-related symptoms and abnormal radiologic findings at the primary or new locations during treatment. Poor drug compliance, drug side effects, and other infections were excluded. ESR, IL-6, and CRP levels were markedly elevated. In addition, this patient showed a rapid and remarkable response to steroids. All of the above suggested a diagnosis of brucellosis-IRIS.

There are many similarities between the pathogenesis of MTB infection and that of brucellosis (10, 11). During MTB infection, multiple MTB components interfere with host cellular functions, inciting specific host immunodeficiency and helping the pathogen evade host innate immunity (12). A similar phenomenon also occurs in *Brucella* infection. For example, the outer membrane protein of *Brucella* can inhibit the production of TNF (13), IL-12 (14), and IFN-β (15); depress T-cell responses; and compromise monocyte/macrophage function, causing temporal immunosuppression (16). Therefore, it can invade multiple organs and often induce chronic infection (17). It is speculated that the mechanism of brucellosis-related IRIS is similar to that of TB-IRIS in HIV-uninfected individuals.

On the basis of previous studies, paradoxical reactions to TB-IRIS in immunocompetent patients have been attributed to immunological causes (6, 18). Antibiotic therapy leads to an apparent reversal of the immunosuppressive state, with phagocytosis of mycobacteria and a rapid onset of local cellular immune responses (5). An overwhelming and exaggerated immune recovery may lead to excessive immunopathological damage at the tissue level.

It is believed that patients with a high bacterial load have a high degree of immunosuppression at the foci of infection. We feel that, in the patient with effective antimicrobial therapy, the bacterial load is reduced, and host immunosuppression is restored, leading to an excessive inflammatory response. In addition, this patient was a young male, and according to TB-IRIS data, young age and male sex are high-risk factors for IRIS (19).

There is no standard treatment for IRIS; some patients experience spontaneous resolution, whereas others require the use of anti-inflammatory drugs, depending on the site and severity (20).

There are no previously reported cases of *Brucella*-related IRIS. This may be because IRIS might be misdiagnosed as superimposed infections, inadequate anti-*Brucella* treatment, or relapse. It is necessary to be aware of the possible occurrence of IRIS in brucellosis patients in clinical practice. Clinical deterioration during antibiotic treatment may be interpreted as treatment failure, leading to the change of antibiotic regimens or the prolongation of their use.

However, our study has some limitations. First, the high level of bacteriological hazard of live *Brucella* did not allow us to perform a drug susceptibility test for isolated *Brucella.* Additionally, we did not further screen the patient for potential immunodeficiency.

In summary, this is the first suspected case report describing paradoxical reactions during the treatment of *Brucella*. The case that we report here demonstrates that IRIS may occur during the treatment of *Brucella* infection. It is urgent to develop a definition of *Brucella*-associated IRIS for accurate diagnosis. The epidemiology, pathophysiology, and risk factors for *Brucella*-associated IRIS need further study.

## Data Availability Statement

The original contributions presented in the study are included in the article/supplementary material. Further inquiries can be directed to the corresponding author.

## Ethics Statement

The studies involving human participants were reviewed and approved by the Shandong University Qilu Hospital human research protection committee. The patients/participants provided their written informed consent to participate in this study.

## Author Contributions

GW conceived of and coordinated the study. Material preparation, data collection and analysis were performed by CQ, DN, SaW, HY and ShW. The first draft of the manuscript was written by CQ. NX and GW edited and revised the manuscript. All authors read and approved the final manuscript.

## Conflict of Interest

The authors declare that the research was conducted in the absence of any commercial or financial relationships that could be construed as a potential conflict of interest.

## Publisher’s Note

All claims expressed in this article are solely those of the authors and do not necessarily represent those of their affiliated organizations, or those of the publisher, the editors and the reviewers. Any product that may be evaluated in this article, or claim that may be made by its manufacturer, is not guaranteed or endorsed by the publisher.
